# The outcome of the retrosigmoid approach in the decompression of vestibular schwannomas – a retrospective cohort study of 60 consecutive cases

**DOI:** 10.25122/jml-2024-0055

**Published:** 2024-04

**Authors:** Hassan Kadri, Mohamad Shehadeh Agha, Raed Abouharb, Rostom Mackieh, Thea Kadri

**Affiliations:** 1 Department of Neurosurgery, Faculty of Medicine, Damascus University, Damascus, Syria; 2 Department of Internal Medicine, Faculty of Medicine, Damascus University, Damascus, Syria; 3 Department of Biology, George Washington University, Washington, USA

**Keywords:** vestibular schwannoma, sudden sensorineural hearing loss, hearing loss, facial weakness, rectosigmoid approach

## Abstract

This multicenter retrospective cohort study aimed to evaluate the effectiveness of the retrosigmoid surgical approach in decompressing vestibular schwannomas, focusing on tumor decompression, neurological function preservation, and postoperative complications. A cohort of 60 patients, operated between 2016 and 2019, was analyzed for age, sex, symptoms, tumor size, surgery duration, complications, mortality, and facial/auditory functions using established criteria. Hearing loss was observed in 80% of patients, mainly progressive, with tumor size emerging as a critical prognostic factor. Facial weakness affected 10% of patients preoperatively; postoperatively, 35% of patients had affected facial nerve function, with 10% exhibiting poor or no facial nerve function, linked to resection extent rather than tumor size. Tinnitus was more prevalent with larger tumors, whereas headaches were common irrespective of size. Balance disorders improved after surgery, especially in case of larger tumors. Functional recovery varied, with 41.67% of patients returning to their previous activity within 4 months, 25% within 4–12 months, and 33.33% remaining inactive. The mortality rate was low at 3.3 %, with two deaths out of 60 patients after surgery. This analysis highlights surgery risks for vestibular schwannomas (e.g., facial nerve decline, tinnitus, headaches), but also emphasizes benefits like improved balance and low mortality. Many patients regain professional activity, stressing the importance of informed treatment decisions for this condition.

## INTRODUCTION

Vestibular schwannomas, also known as acoustic neuromas, are tumors that typically develop from the vestibulocochlear nerve and are the most common type of mass found in the cerebellopontine angle, accounting for 75–90% of cases [1–3], and 8–15% of all primary intracranial tumors. In 95% of cases, these tumors are solitary and sporadic. However, bilateral vestibular schwannomas are strongly associated with neurofibromatosis type 2, although they can also occur in the absence of other neurofibromatosis type 2 symptoms. Vestibular schwannomas are most commonly diagnosed in people aged 40–60 years, but they can also occur in children.

The most common symptoms of vestibular schwannomas are adult-onset hearing loss and non-pulsatile tinnitus, but in some cases, patients may not experience any symptoms until the tumor grows and starts to cause mass effect. This can lead to symptoms such as cranial nerve dysfunction or hydrocephalus owing to compression of the fourth ventricle. The diagnosis is established by magnetic resonance imaging in addition to auditory and clinical evaluation [4,5].

The objective of this study was to analyze the outcomes related to the retrosigmoid surgical approach in decompressing vestibular schwannomas, specifically its efficacy concerning tumor decompression, the preservation of neurological function, and the occurrence of postoperative complications.

## MATERIAL AND METHODS

We conducted a multicenter, retrospective cohort study involving 60 patients with vestibular nerve sheath tumors who underwent surgical intervention at the Department of Neurosurgery of the Faculty of Medicine, Damascus University between 1 January 2016 and 31 December 2019. The collected data included age, sex, symptoms upon admission, tumor size, surgery period, length of hospital stay, radiological investigations, surgical outcome, short- and medium-term complications after surgery, and mortality.

The inclusion criteria were: patients with unilateral eighth nerve tumors, a tumor diameter of ≥15 mm, confirmed schwannoma histopathology, and patients operated on by retrosigmoid approach. Patients under 20 years of age, with a tumor diameter of <15 mm, bilateral tumors, tumors located within the internal auditory meatus only, or whose data could not be completed through the patient file kept in the hospital archive were excluded. Patients who died before surgery or were not eligible for surgery, had small tumors with minimal symptoms and were under observation, or had associated intracranial tumors affecting the outcome were also excluded. We aimed to follow up with as many cases as possible for a period of 6 months to 3 years after surgery. The average follow-up time was 17 months.

The evaluation of facial nerve function before and after surgery was conducted using the House–Brackmann scale [6]. Auditory function was assessed using the pure tone audiometry test and speech discrimination score. Based on the results of these tests, patients were categorized into four grades according to their level of auditory function, with grade A representing the best function and grade D representing almost complete absence of function. This classification system has been approved by the American Academy of Otolaryngology-Head and Neck Surgery (AAO-HNS). The study used a questionnaire to gather the necessary information [7,8].

All patients had magnetic resonance imaging with gadolinium injection performed before surgery and many times after surgery during the follow-up period.

### Statistical analysis

Descriptive statistics (mean ± s.d., frequency, and percentage) were used to summarize the characteristics of the patient cohort, such as age at diagnosis, sex distribution, tumor location, tumor size distribution, and the period between symptom onset and final diagnosis. Frequency distributions were used to present the distribution of patients based on various factors, such as age groups, tumor location (left or right side), tumor size categories, and the occurrence of symptoms. Percentages were used to express the proportion of patients within each category, providing a clear overview of the cohort’s characteristics. The chi-squared test and cross-tabulation were used for comparative analysis. A *P* value of ≤0.05 was considered statistically significant.

## RESULTS

The mean age at diagnosis was 55.6 years (range, 20–80 years). Of the 60 cases, 6 (10%) were aged between 20 and 39 years, 42 (70%) were between 40 and 59 years, and 12 (20%) were between 60 and 80 years (Table 1). There were 24 male patients (40%) and 36 female patients (60%), resulting in a female-to-male ratio of 1.5:1.

**Table 1:** Patient characteristics at admission

Variable	*n* (%)
**Age, years**	
20–29	6 (10%)
40–59	42 (70%)
60–80	12 (20%)
**Side of tumor**	
Right	27 (45%)
Left	33 (55%)
**Tumor diameter**	
15–25 mm	15 (25%)
>25 mm	45 (75%)

The patients were divided into two groups based on the location of the tumor: 27 cases (45%) had tumors on the right side, and 33 cases (55%) had tumors on the left side (Table 1). Tumor size was determined using magnetic resonance imaging with gadolinium injection, with a radiological diameter ranging from 15 to 50 mm. No case was reported in which the tumor was entirely contained within the internal auditory canal. The patients were classified into two groups based on tumor size, which was measured according to the maximum extrameatal diameter. Of the 60 patients, 15 (25%) had medium-sized tumors (15–25 mm), and 45 (75%) had large tumors (>25 mm) (Table 1).

The period between symptom onset and final diagnosis varied from 3 months to 10 years. In total, 22 patients (36.67%) were diagnosed more than 4 years, nine patients (15%) were diagnosed between 2 and 4 years, 14 patients (23.33%) were diagnosed between 1 and 2 years, and 15 patients (25%) were diagnosed within a year after symptom onset.

The main symptoms at admission are summarized in Table 2. The most common clinical presentation was unilateral hearing loss in 48 patients (80%), which developed gradually and progressively in 96% of cases, except for two patients (4%) who had sudden hearing loss. Tinnitus was reported in 34 cases (56.67%), and balance disorder in 33 cases (55%). Facial weakness was observed in 6 patients (10%), of whom two had moderate weakness of degree 3, whereas the rest had severe weakness of degree 4 or more. Facial numbness and paresthesia were reported in eight cases (13.33%), headache in 18 cases (30%), ear pain in three cases (5%), and dysphagia or hoarseness in two cases (3.33%).

**Table 2:** Main symptoms at admission

Symptom	*n* (%)
Unilateral hearing loss	48 (80%)
Tinnitus	34 (56.67%)
Balance disorder	33 (55%)
Facial weakness	6 (10%)
Facial numbness and paresthesia	8 (13.33%)
Ear pain	2 (5%)
Dysphagia or hoarseness	2 (3.33%)
Headache	18 (30%)

All tumors were enhanced with contrast material. In total, 37 tumors (61.67%) were homogeneously enhanced, 17 tumors (28.33%) were heterogeneously enhanced, and 6 tumors (10%) were cystic.

Surgical treatment was undertaken in all cases; 52 patients (86.67%) underwent total macroscopic resection of the tumor (total and near total resection), and 8 patients (13.33%) underwent subtotal resection. The reasons for subtotal resection of the tumor were severe tumor adhesion to the facial nerve in five patients, adhesion to the brain stem in two patients, and adhesion to both the facial nerve and brain stem in one patient. The structure of the facial nerve was preserved during surgery in 52 patients (86.67%), whereas dissection and excision of the tumor resulted in sacrificing the facial nerve in eight patients (13.33%).

As far as complications after surgery are concerned, cerebrospinal fluid leakage from the wound occurred in nine patients (15%), infarction of the cerebral hemisphere occurred in one patient (1.67%), and posterior fossa hemorrhage occurred in two patients (3.33%). Meningitis occurred in four patients (6.67%) and was treated with appropriate antibiotics based on culture and sensitivity results, leading to clinical and laboratory improvement. Hearing loss was the most common complication, occurring in 53 patients (91%). Tinnitus occurred in 15 patients (25%), balance disorder occurred in seven patients (11.67%), and facial weakness (i.e., a score of 3 or higher on the House–Brackmann scale) occurred in 21 patients (35%). After surgery, good facial nerve function (grade 1 or 2) was observed in 39 patients (65%), moderate function (grade 3) was observed in 15 patients (25%), and poor to non-existent function (grade 4 to 6) was observed in six patients (10%). Headache occurred in 26 patients (43.33%). Dysphagia or hoarseness (injuries at the level of the lower cranial nerves) occurred in two patients (5%).

The postoperative mortality rate was 3.33% (two patients). One patient died approximately one month after surgery owing to aspiration pneumonia resulting from the inability to swallow due to paralysis of the lower cranial nerves. Another patient died owing to pulmonary embolism 5 days after surgery. Immediate postoperative complications are summarized in Table 3.

**Table 3:** Immediate postoperative complications

Complication	*n* (%)
Cerebrospinal fluid leak	9 (15%)
Cerebellar ischemic lesion	1 (1.67%0
Bleeding	2 (3.33%)
Meningitis	4 (6.675%)
Hearing loss	55 (91%)
Tinnitus	15 (25%)
Balance disorder	7 (11.67%)
Facial weakness	21 (35%)
Headache	26 (43.33%)
Dysphagia or hoarseness	3 (5%)

## DISCUSSION

In this study, the clinical symptoms of patients, including hearing loss, facial weakness, tinnitus, balance disorder, and headache, were compared before and after surgery. The results showed that some symptoms improved and others worsened after surgery, and the size of the tumor and the degree of surgical removal were identified as factors affecting the results of surgery.

### Hearing loss

**Figure 1:**
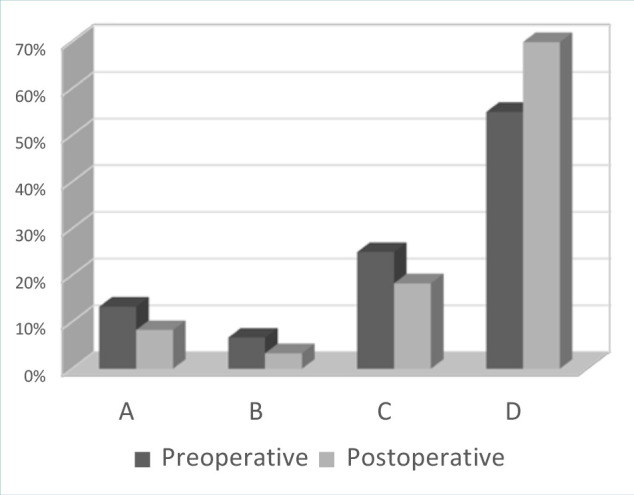
Hearing loss before and after surgery according to the AAO-HNS classification

Hearing loss was classified according to the American Academy of Otolaryngology – Head and Neck Surgery Foundation (AAO-HNS) hearing classification system, which defines hearing loss as anything less than grade B. Hearing loss is the most commonly encountered symptom in patients with vestibular schwannoma, occurring in 80% of patients [9] and may be related to the size of the tumor [10,11]. It has also been reported that hearing loss appears progressively in more than 90% of cases, but it can also occur suddenly [9–12]. Interestingly, some authors report that sudden hearing loss due to vestibular schwannoma accounts for 4% of all cases of sudden sensorineural hearing loss [13]. In our study, 48 patients (80%) had hearing loss, which occurred progressively in 96% and suddenly in 4% (Figure 1). Of these 48 patients, 33 had complete hearing loss (grade D), 15 patients had grade C auditory function, and 4 patients had grade B auditory function. Hariti reported the preservation of hearing function in 2 out of 49 patients with large tumors [14]. At the same time, he reported that the majority of patients were functionally independent. Other authors found that serviceable hearing (grade A or B) was preserved in 70.4% of patients [15]. Unfortunately, this preservation of acoustic function could deteriorate with time [16,17]. In our study, 55 patients (91%) presented with hearing deficits after surgery, grade D hearing loss occurring in 42 patients (70%), grade C in 11 patients (18.33%), grade B in 2 patients (3.33%).

Both the Hannover size classification and millimetric measurement are used to determine the size of the vestibular schwannoma [18,19]. Tumor size has an important role in the preservation of hearing function [20]. Among the patients with tumors between 15 and 25 mm, five had grade A hearing, one had grade B hearing, five had grade C hearing, and four had grade D hearing. Among the patients with tumors larger than 25 mm, one had grade B hearing, six had grade C hearing, and 38 had grade D auditory function. It was noted that in case of large tumors the surgical intervention was associated with a high rate of cochlear nerve injury and thrombosis of the internal auditory artery, both of which are associated with permanent hearing loss.

Our findings suggest that the most important prognostic factor for predicting the audiological outcome of surgery is the size of the tumor. This is consistent with the findings of previous studies [21].

In another study, gross total resection was achieved in 83% of the cases, near total resection was achieved in 15% of the cases, and subtotal resection was performed in 2% of the cases [14]. In our study, 52 patients (86.67%) underwent total macroscopic resection of the tumor (total and near total resection), and eight patients (13.33%) underwent subtotal resection.

### Facial weakness

**Figure 2:**
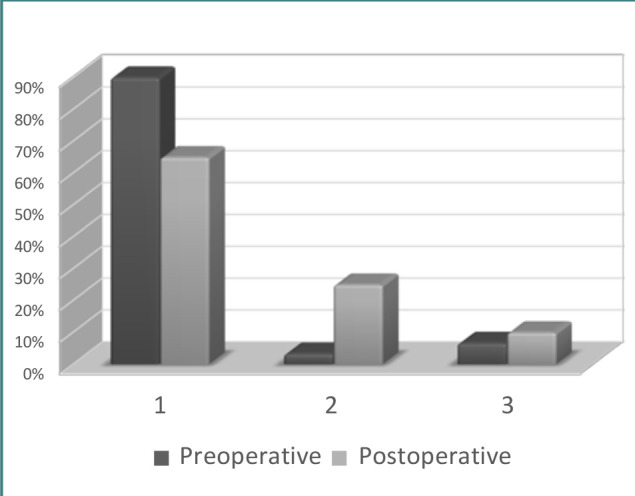
Functionality of the facial nerve before and after surgery according to the House–Brackmann scale

In our study, preoperative facial weakness was evident in six (10%) of the 60 patients examined (Figure 2), from which two had moderate function (House–Brackmann grade 3) and four demonstrated poor to no function (House–Brackmann grade 4 to 6). According to previous research, large vestibular schwannomas may affect the trigeminal nerve in 40–80% of patients and the facial nerve in 10–20% of patients [22–25].

The recovery of facial function should be evaluated in the first 6 months following surgery. Studies have shown that the preservation of facial nerve function was achieved in up to 78% of vestibular schwannomas operated using the retrosigmoid approach [26,27].

A decline in facial nerve function was observed after the surgery; 39 patients (65%) had good function (House–Brackmann grade 1 or 2), 15 patients (25%) had moderate function, and six patients (10%) had poor to no function (House–Brackmann grade 4 to 6). During the operation, the facial nerve structure was preserved in 54 patients (90%), and dissection and tumor removal resulted in sacrificing the facial nerve in six patients (10%) to achieve complete eradication of the tumor.

In the group that underwent subtotal tumor resection, facial nerve function was moderate in one patient (12.5%) and good in seven patients (87.5%). Meanwhile, in the group that underwent total tumor removal, facial nerve function was poor or non-existent in six patients (11.54%), moderate in 14 patients (26.92%), and good in 32 patients (61.54%). Interestingly, we found that poor facial nerve function after surgery was not linked to tumor size but rather to the extent of resection. Complete tumor resection is often associated with poor facial nerve function [28], whereas subtotal resection is more likely to preserve relatively good function, even in cases involving large tumors. Some researchers reported that 69–80% of patients had excellent facial nerve function (grade 1–2) after surgery, with a fair outcome in 20–31% of cases [14,29]. However, it should be noted that preservation of the anatomical integrity of the facial nerve does not guarantee normal function after surgery because of various factors such as inflammation, vascular spasm, and viral reactivation [12,29]. In fact, Rinaldi *et al*. found that even with preservation of the facial nerve in 97% of their patients, only 77.4% demonstrated good functionality [30].

### Tinnitus

Preoperative tinnitus has been reported in 6.3–57% of patients [9,25,31]. In our study, 34 out of 60 patients (56.67%) had tinnitus before surgery. Postoperatively, 25 of these patients (41.67%) saw an improvement in their symptoms, whereas nine continued to experience tinnitus. Additionally, 6 patients (10%) developed tinnitus after surgery. In total, 15 patients (25%) suffered from postoperative tinnitus.

Although tinnitus is not a size-dependent feature of vestibular schwannoma [24,32], we observed that it occurred more frequently among patients with tumors larger than 25 mm, and it improved in most patients after surgery. Among patients with tumors of 15–25 mm, six patients (40%) suffered from tinnitus before surgery and three patients (20%) after surgery. Among patients with tumors larger than 25 mm, 28 patients (62.22%) suffered from tinnitus before surgery and 12 patients (26.67%) after surgery.

### Headache

Studies suggest that the preoperative incidence of headache is 2–37%, being more commonly encountered in case of larger tumors [9,31]. A review of the literature indicates that postoperative headache is more commonly associated with smaller-sized tumors, and that the retrosigmoid approach with craniotomy results in lower rates of postoperative headache compared to the same approach with craniectomy [33]. However, some systematic reviews reported contrasting results regarding the frequency of headaches and the optimal approach for resecting cerebellopontine angle tumors [34]. Other studies found that tumor size and treatment modality have no impact on headache [35].

In our study, 18 patients (30%) had headaches, of whom six showed improvement after surgery and 12 continued to experience headaches. Among the patients with tumors of 15–25 mm, four patients (26.67%) had headaches before surgery and five patients (33.33%) after surgery. Among patients with tumors larger than 25 mm, 14 patients suffered from headaches before surgery (31.11%) and 21 patients (46.67%) after surgery. Patients with larger tumors had a higher incidence of headaches, and after surgery, most patients experienced worsened headaches with some showing little improvement.

### Balance disorder

The preoperative incidence of balance disorder has been reported as 3.8–44.6% [13,36,37], and gait instability in large tumors was reported to occur in 30–50% of patients owing to brain stem compression [22,26]. In our study, preoperatively 33 patients (55%) had a balance disorder, of whom 27 improved after surgery, and one developed a balance disorder postoperatively, resulting in seven patients (11.67%) experiencing balance disorder after surgery. Among patients with tumors of 15–25 mm, seven (46.67%) experienced balance disorder before surgery and one patient (6.67%) after surgery. Among patients with tumors larger than 25 mm, 26 (57.78%) experienced balance disorder before surgery and six patients (13.33%) after surgery. Overall, patients showed an improvement in balance disorder after surgery, with a greater benefit observed in those with larger tumors.

### Quality of life

Facial palsy can significantly affect a patient’s quality of life, with their perception of their own handicap having a major role in its effects. Interestingly, the severity of facial impairment may not always correlate directly with the level of psychological distress and social function [36,37]. Similarly, unilateral hearing loss can also greatly impact a patient’s quality of life, as it reduces their ability to localize sounds and may result in slight social dysfunction [38].

In our study, 25 patients (41.67%) regained their activity in less than 4 months after surgery, 15 patients (25%) regained their activity between 4 and 12 months after surgery, and 20 patients (33.33%) did not regain their previous activity. The main factors affecting the quality of life of patients were hearing loss (affecting speech), balance disorder (impairing physical activities), and facial weakness (causing social embarrassment). Of the 35 patients who were employed before surgery, 25 have returned to work, two-thirds within 4 months and one-third within 12 months. Five patients (14.29%) voluntarily resigned from their job, three patients (8.57%) were unable to work after surgery, and two patients (5.71%) underwent vocational rehabilitation with job exchange.

### Death

In an analysis of a large group of 4,886 patients who underwent surgery for vestibular schwannoma, the postoperative mortality rate was found to be 0.5% [39]. Another study also reported a 0.5% postoperative mortality rate, owing to bleeding, albeit from a much smaller group of 49 patients [14]. In our study, two out of 60 patients (3.3%) died after surgery.

## CONCLUSION

In conclusion, the analysis of 60 cases of vestibular schwannoma operated on by retrosigmoid approach showed that the surgery led to a decline in facial nerve function, even when the anatomical integrity of the nerve was preserved. Tinnitus was more likely to occur in larger tumors, and patients with larger tumors also had a higher incidence of headaches both before and after surgery. Although most patients experienced worsened headaches after surgery, some showed little improvement. On a positive note, there was an improvement in balance disorder after surgery, and the mortality rate was low at 0.06%. Additionally, 41.67% of patients regained their professional activity after surgery. These findings underscore the importance of carefully considering the risks and benefits of this surgical approach when deciding on a treatment plan for vestibular schwannoma.
